# Core Values at Work—Essential Elements of a Healthy Workplace

**DOI:** 10.3390/ijerph191912505

**Published:** 2022-09-30

**Authors:** Michel Guillemin, Robin Nicholas

**Affiliations:** 1 Faculty of Biology and Medicine University of Lausanne, 1011 Lausanne, Switzerland; 2 Robin Nicholas Communications, Santa Fe, NM 87501, USA

**Keywords:** core values, occupational health, wellbeing, dignity

## Abstract

This paper explores core values at work—those values that give meaning to people’s lives and their work, that allow each person to experience their work with passion, commitment, dignity, and respect. Though core values may appear to be simple and obvious, supporting them at work can be more complex and difficult than expected. These values are not only ethical and moral, but also social and cultural; they are intrinsically related to the same factors that promote health across the globe, including family, community, and work. Three seminars outlined the nature and importance of core values within occupational health and well-being; these sessions were held during the International Commission on Occupational Health (ICOH) conferences between 2015 and 2020. A non-exhaustive, literature review explored these findings further. Perspectives from international, national, and local occupational health programs have begun to demonstrate how core values underpin workers’ well-being. These essential core values impact worker health positively when they are included and respected, and negatively when they are missing or corrupted. Within occupational safety and health (OSH), people’s awareness of these values and their importance at work is now clearly emerging, offering opportunities to honor and protect each worker and help them to experience their core values through their work.

## 1. Introduction

Work is one of the most important human activities that contributes to shape and develop society. First, work must provide the means to food, shelter, clothing, health, and the fulfilling of responsibilities. However, once these essential requirements are met, work has the potential to serve other human needs, including one’s core values. Work based on core values gives sense and meaning to it and aligns with the individual and the values that drive people’s lives. These values are not only ethical and moral, but also social and cultural; they are intrinsically related to factors that promote health across the globe, including family, community, dignity, and respect [1]. However, the question remains—“How can people bring these core values to their everyday experience at work?”.

Core values are those essential values by which a person measures their life, even when those values are diminished, denied, or removed. Frank Rose, drawing upon the work of Philip Tetlock and others, describes core values as moral imperatives that people are unwilling to compromise [2]. This can include family, community, dignity, and respect, as well as the core values people experience at work. When people are true to their core values personally and professionally, they remain true to themselves. This basic summary of core values parallels multiple disciplines that intersect the workplace, including education, management, business, psychology, philosophy, etc. 

The emphasis for this paper is on the personal, core values of workers. This is not about company vision statements or leadership strategies, though these certainly overlap. Simply, the emphasis here is not upon the company but upon the individual and the values through which they experience their work.

It becomes a challenge to define these values in great depth because they are based upon human and often personal experiences—experiences that underpin people’s lives, including their lives at work, and subsequently, their health at work. In many ways, these are intangible assets that require going beyond objective and quantified evaluations to also include subjective tools to approach people’s subjective experience of their values. Our focus here will be on those core values that people share and help bring them together. Though workers may express these values differently across different cultures, the similarity and importance of these values remain the same. We recognize that these positive and ethical dimensions do not include money, power, control, renown, etc. as primary values. However, our intention with this paper is to keep the concept of core values within its positive, human, and constructive dimensions, and consider how these qualities affect people at work.

As people work, occupational safety and health (OSH) strives not only to prevent the negative effects of work on people’s health, safety, and work ability, but also to build the healthy work conditions and work culture that promote good health, well-being, motivation, and job satisfaction. Core values are an essential element of that healthy work culture. To pursue this, the International Commission on Occupational Health (ICOH) held three special sessions dedicated to core values during conferences in Seoul (2015), Dublin (2018), and Mumbai (2020). The Scientific Committee on Education and Training in OH observed this growing interest in core values and created in 2020, the new Working Group—Core Values at Work.

The first two sessions were entitled, Work and Spirituality, and the third one, Core Values at Work. The word spirituality was offered—not in a religious sense—but as a way to view the whole person, going beyond the material aspects of people’s lives. It embraces workers’ deep feelings and beliefs that carry them through the day—the meaning of work, the core values of respect and dignity, and people’s relationships with family, friends, and community. However, because some cultures interpret the word spirituality differently, the title of the sessions was changed to Core Values at Work, while still addressing how each individual experiences themself through their life and work.

This paper will explore how workers’ core values can act as key elements of a healthy workplace. This approach is already emerging. As one example, the World Health Organization (WHO) developed a model to illustrate the different avenues of influence, process, and core principles of a healthy workplace, and placed ethics and values at the very center of the process [3]. These essential values need to be better understood within the context of global health at work [4] so that the elements that make up worker well-being are clearly identified. As noted earlier, these qualities are intangible assets where there are no metrics to measure or evaluate them. This creates a challenge—one that this paper will explore, as well as offer possible tools and solutions.

This exploration of values begins with what the three ICOH special sessions revealed, together with perspectives from a non-exhaustive review of relevant studies that illustrate holistic and human dimensions of core values at work. The specific OSH focus will be based upon (1) the experiences of workers within their own work culture; (2) new ideas and innovations in research, and education; (3) global and regional examples already in practice; and (4) specific, interdisciplinary issues related to well-being, mental health, culture, and leadership. The results will then be discussed within the context of society’s evolution as it considers the meaning of work in today’s changing environment, emerging new approaches to governance and leadership, and the promises and dysfunctions of a new way of being at work.

## 2. Materials and Methods

The methods of this study evolved to explore how core values at work are lived and practiced from multiple perspectives. The three ICOH special sessions from 2015 to 2020 provided the first steps to collect information and build the main concept of core values at work.

The first two sessions were panel presentations. Speakers were invited with a concerted effort to bring together a broad spectrum of international, national, and local organizations, and across a variety of disciplines, including occupational health, industrial hygiene, management, communications, and others. Each session was open to all ICOH Conference attendees interested in attending the session. Following each presentation, attendees offered questions and comments for discussion. The third session was structured as a workshop, where participants engaged the speakers in the importance that values hold in the workplace, and how core values at work could proceed. The theme for all three sessions remained the same; however, the speakers were selected to broaden the scope in each subsequent workshop within the limits in time and space offered by the Conference organizers.

At all three conferences shown in Table 1, presenters gathered from multiple countries, cultures, and backgrounds, providing wide-ranging perspectives. The first session started with the dimensions of work that address the whole person—including people’s fundamental values, needs, and aspirations. This approach was explored further through examples from international, national, regional, and individual perspectives. The second session considered how culture and the potential, positive effects of work influence these fundamental values and how they are practiced. In the third session, the new working group, Core Values at Work, explored new directions to learn how people experience their work and what tools they can use to live their values at work.

Based upon the insights gained through these three sessions, a non-exhaustive review of the professional literature provided a means to identify specific examples of how core values are explored across different private and public organizations, across diverse cultures, and amongst many disciplines and schools of thought. The literature review focused on how core values are becoming a more relevant topic in workplace health and the meaning of work. We did not carry out a systematic review of literature through the usual data banks, but rather a non-exhaustive review based on our experience and our networks of colleagues and peers. We recognized that there are numerous discussions and research in different areas that relate to core values. However, it is impossible to cite or reference them all. By acknowledging that this is a non-exhaustive search, we intend to provide specific examples within the literature that illustrate key points about the workers and their human values.

## 3. Results

### 3.1. A Global Exploration—ICOH Special Sessions

The name Core Values at Work implies an entire spectrum from each worker’s personal experience of their values to the organizations that provide the work itself.

The first ICOH special session explored this spectrum from the workers to international organizations. These included outlooks from international organizations such as the WHO and the Pan American Health Organization (PAHO), from national organizations such as Total Worker Health™ in the USA, from academic institutions and specific disciplines such as ergonomics and well-being at work, and from OSH communications that consider the full scope of why people work [5].

The second ICOH session worked to go beyond the primary OSH focus on preventing disease and illness, to a comprehensive vision of work that considers the potential positive effects of work on a person’s health. The result is a more integrated approach to workers’ health and well-being, strongly related to fundamental human values. Participants provided special examples from South Africa and India. In South Africa, the philosophy of *ubuntu* presents the essence of what it means to be human, promoting the virtue of compassion which embodies the greater good of growing together and caring for one another. A study was also presented from India of current work to help sewage workers where their work is regarded as discriminatory and stigmatizing, resulting in hiding community and identity and feeling a loss of dignity. Moreover, for workers with personal challenges such as cancer and chronic disease, the importance of the quality of a working life was related to key issues such as individual perceptions of work, social structure, the work environment, and characteristics of the work organization itself. Through all these contexts, mindfulness was explored as a useful tool to improve social, mental, physical, and holistic well-being. In addition, recognizing people’s experience of work offered an approach to investigate ways for people to explore and understand themselves and the values that strengthen their self-excellence, social relationships, and personal well-being [6].

The scope of the third session and workshop was to help build awareness about the importance of core values at work, to explore tools for workers to integrate personal values into their work. The workshop also emphasized the responsibilities of leaders to take into account each worker’s needs and aspirations. To be effective for people and the businesses where they work, these values must be shared and implemented across the whole organization where decision-making, information-sharing, communication and culture are aligned with workers’ values. If these values are not shared, the human element is gradually pushed out of the workplace, resulting in less dignity and respect, less efficiency, and less safety and health. During the session, a comparison between the Australian and Asian approaches revealed how to create a synergy for OSH professionals who have worked across different cultures. In this workshop, participants helped define personal core values while identifying challenges to implementing this human approach within a society where profit and productivity are often the priorities. Everyone agreed on the importance of continuing this work [7].

Beyond these three sessions, the non-exhaustive review of the professional literature provided a means to identify how core values are explored across different private and public organizations, across different cultures, and amongst many disciplines and schools of thought.

### 3.2. Core Values and the Individual

Though the term core values is not always literally identified, many approaches to worker health and safety effectively address core values. Worker well-being encompasses core values by its very nature, because it embraces the whole person, including their physical, psychological, and emotional health and safety. Räsänen and Anttonen presented a perspective of well-being that easily applies to a healthy workplace where workers flourish and achieve their full potential [8]. Schulte and Vainio, Waddell and Burton, and others have also presented the breadth of worker well-being at work [9,10]. Wright and Cropanzano [11] have taken this approach further, showing that people who experience well-being bring more to their work.

Similarly, the field of salutogenesis offers a path that parallels core values and worker well-being by going beyond disease prevention to promoting complete health of the whole person. Again, within this holistic approach to health, core values provide an essential element in creating healthy and fulfilling work [12].

The psychology of work looks at the thoughts and emotions of workers and their resulting behaviors. Recent work in behavioral studies and neuroscience has shown how humans respond to work. The classical works of Damasio and Maslow have offered insight into human behavior, while Wrzesniewski and Schwartz, Philips and Edwards, and others looked at the specific values of people at work [13,14,15,16].

Exploring people’s cultures has expanded how people view themselves within their own work culture. International organizations such as the United Nations Educational, Scientific and Cultural Organization (UNESCO) considered cultures’ influence across the globe. Other approaches by Mkhize and others have explored how specific worldviews affect work [17,18].

Core values have been shown through multiple surveys to be an important factor in what makes a job meaningful. Two, large surveys in the US and the UK found that jobs that offer a degree of professional independence and make a social contribution are the most meaningful and worthwhile [19]. In addition, workers have described values-related aspirations for their job—what they desire most for their job. These include work that is meaningful, has supportive leadership, offers a positive work culture, and brings good, social relationships [20].

Workers’ need for a more meaningful job has incited companies to look for new paradigms in governance and leadership. In this context, two visionary economists, Barrett [21] and Laloux [22] have investigated new management models based on organizations that have a soul, in other words, that are based on core values. They opened new avenues for prevention of work-related diseases and for promoting well-being at work, not only for workers but also for their leaders [4]. Reicher and others have outlined how values leadership helps to bring these values to the workplace [23].

### 3.3. Core Values Supported within Organizations and OSH

Today, the meaning of work is changing rapidly for several reasons. Technology, artificial intelligence, and global use of online social networks continue to influence how people work. Workers are holding multiple jobs, requiring them to rely more and more on digital platforms, and converting to remote work, especially since the Covid pandemic. All these changes are causing people to reexamine their experience and perspective of work and the role their core values play.

Within this evolving environment, the global approach of OSH continues with a wide range of partners, including workers, leaders, decision-makers, politicians, and more. As the role of these players becomes more important [24], core values provide an essential element as a holistic approach by international, national, and local organizations. This is exemplified by the WHO [3], The Vision Zero program in the International Social Security Association (ISSA) [25], and the Total Worker Health™ program in the United States [26]. As these programs progress, they have revealed the need for greater awareness and training as these values emerge as part of worker health [27].

Fundamental principles are already required for health and safety professionals, such as those outlined in the ICOH Code of Ethics [28]. However, core values are often not identified as essential elements within OSH programs. Still, some research has been conducted [29] and identified the need to include values when training OSH professionals. Similarly, Zwetsloot and others have explored how institutional and company values can support worker core values as part of their health, safety, and well-being at work [30]. A good way to promote awareness and progress is to give more visibility to companies and enterprises where core values are promoted within their work culture.

As with any positive, well-intentioned work, there are challenges to values-based management while creating a positive workplace that recognizes and promotes worker values, building mutual trust between partners, and placing values into practice. Even with good intentions, mistakes can happen. People can make unethical decisions without realizing it [31], or they can misuse core values to simply pressure workers to work harder [32]. Social responsibility is an important quality of conscientious companies when it is genuine [33]. However, when used only as a marketing tool, problems arise [34]. To meet these challenges, companies must remain vigilant and promote core values as a natural part of their work culture [34].

## 4. Discussion

As described earlier, through the three ICOH sessions and the literature review, core values at work implies the full spectrum from the individual worker to the organizations that promote health, safety, and well-being, to the companies and agencies that provide the actual work and support their workers.

For people to function and contribute all of themselves at work, the workplace needs to support them as whole, human beings. This cannot be accomplished by objective, quantifiable, and logistical practices alone. People also need and want to bring their passion, conviction, and dedication to their work. These are the personal experiences, dynamics, and values that bring people to their work—including their safe and productive work. Current work environments often emphasize logistical, logical, and linear approaches. These are essential but they are insufficient by themselves; they do not represent all that a person is because people are much more than their rational minds. Simply, each person needs to bring all of themself to work.

### 4.1. Core Values and the Individual

Ultimately, core values at work must support the individual. It is their core values that form the foundation of the work culture. Traditionally, workplace health and safety has looked at the technical and quantifiable aspects of safety—What are the risks? What work practices are needed? The answer is usually objective, expressed in terms of work procedures, equipment, policies, and regulations.

However, before this, there is the initial question—Why are people being safe? Here, the answer is subjective; it is about family, relationships, and emotions—and it is about values. This leads to the essential, core value that forms the foundation of OSH: People value life and the quality of life. It is for this reason that the WHO places values at the very center of global health [2]. Simply, everyone’s life is to be celebrated and protected. This includes not only physical life, but also the qualities of life, such as self-respect, dignity, and personal excellence. All these values contribute to a person’s inner health, which then contribute to their outer health. Without that inner health, people’s outer health and well-being are incomplete [35].

#### 4.1.1. The Whole Person and Well-Being

As part of well-being, core values are basic to life, including people’s work life. Perhaps Martin Buber described it best, “I think no human can give more than this…making life possible for the other” [36]. This is why occupational safety and health is so important, when it encompasses the entire person.

When work supports the whole person, it supports their entire well-being. Schulte and Vainio described how well-being goes beyond physical health to include dimensions of material, social, emotional, and personal development. An individual’s well-being does not exist on its own, nor is it isolated within the workplace; but rather it exists within this broader social context and influences people’s satisfaction with work and life. [9] Waddell and Burton pointed out that well-being is the subjective state of being healthy, happy, contented, comfortable, and satisfied with one’s life [10]. Räsänen and Anttonen offered a perspective that reaches the breadth necessary for this discussion, that well-being is the flourishing of employees so that they can achieve their full potential for both their own benefit and that of the organization [8]. Wright and Cropanzano described a similar dynamic; people who experience well-being bring more to their work [11], by providing insight and contributing to something larger than themselves [37].

#### 4.1.2. The Psychology and Emotions of Work

As much as people may think that work is objective, key emotions underly their good work. Dedication to their work and passion for their work are major, emotional dynamics that must be protected and nurtured by the workplace. The neuroscientist, Antonio Damasio, defined this dynamic well when he described people—“We are not thinking machines. We are feeling machines that think.” [13] People are wired to respond to their emotions, so their emotional commitment at work becomes essential. Ultimately, safety programs must be engaged and implemented by human beings who are much more than intellect. Each person is a complex combination of mind, body, emotions, intuition, relationships, culture, and values; and as a whole person, each person responds to the values of respect, dignity, and well-being at work and throughout their lives [38].

**Figure 1:**
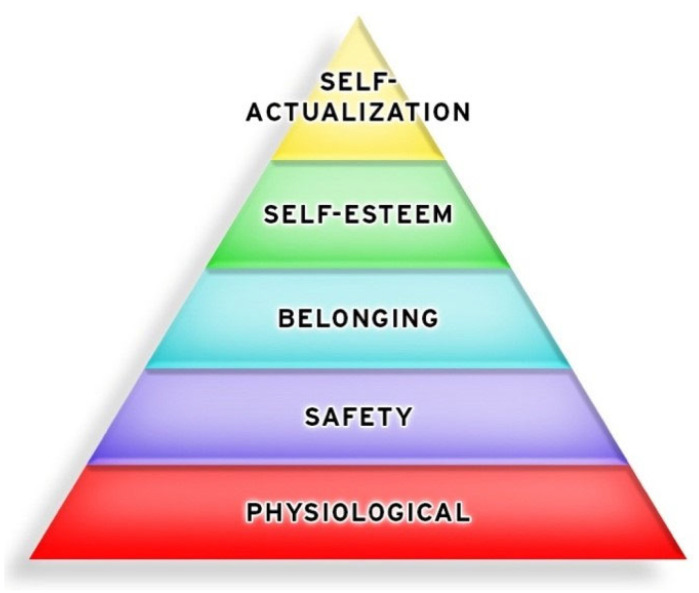
The misused pyramid of Maslow’s hierarchy of needs. This pyramid is often used in presentations, but is misinterpreted and over-simplified as a rigid, linear process.

Advances in psychology have shown how human needs and values impact lives. In 1943, Maslow proposed a hierarchy of needs [14] that offers another way of looking at human values. Physiological and safety needs mirror people’s value of life. The remaining needs of belonging, self-esteem and self-actualization reflect how people value quality of life. Maslow was concerned how large organizations can suppress individual expressions. To counter this, he saw his hierarchy of needs as a means for people to fulfill their essential values—individual creativity, expression, growth, and fulfillment [39]. Unfortunately, others have misrepresented and oversimplified this hierarchy as a layered-pyramid and a rigid, linear process, as shown in Figure 1.

In this misrepresentation, a person must first meet their physiological needs to survive. Then, only after physical needs are met completely can a person move on to the next level and secure their safety. Additionally, after safety is satisfied, a person can supposedly move onto a sense of belonging, self-esteem, and finally self-actualization. The implication here is that a person must satisfy a lower level completely in order to move on to the next higher level. However, people and life do not work that way.

**Figure 2:**
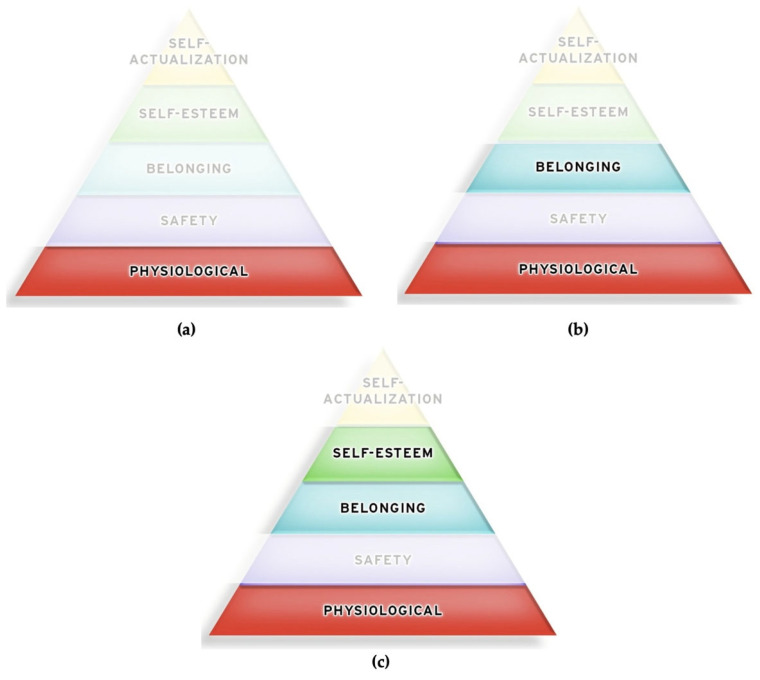
Experiencing multiple needs at the same time. (**a**) A person may be trying to meet their physical needs to survive; (**b**) At the same time, the person wants to take care of their family, feeling a sense of belonging; (**c**) That person can also feel a loss of self-esteem and dignity because they cannot take care of their family the way they want to.

As shown in Figure 2, a worker may be struggling physically to survive, while also struggling to take care of their family. At the same time, because they cannot take care of their family the way they want to, they may feel a loss of dignity. Thus, an individual can experience several needs and values at the same time, and Maslow confirmed this in his work. It is important to remember that Maslow never represented his hierarchy as a pyramid; someone else did that [39].

**Figure 3:**
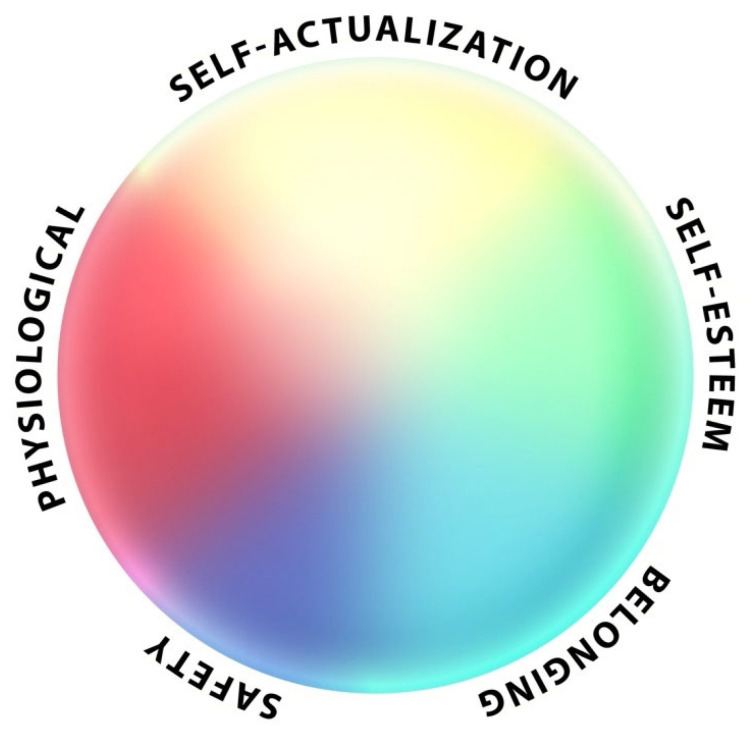
A sphere as a different way to represent multiple needs. A sphere demonstrates how a person can experience multiple needs at the same time. The boundaries between needs are fluid and adaptable.

As a possible correction, there is another option to represent these multiple needs. As shown in Figure 3, instead of a pyramid, a sphere can represent the multiple needs that a person may experience at the same time. Here, the boundaries between different needs are not absolute, they are blurred and fluid. As a person copes with multiple needs, their experience moves closer to the center of the sphere [40].

**Figure 4:**
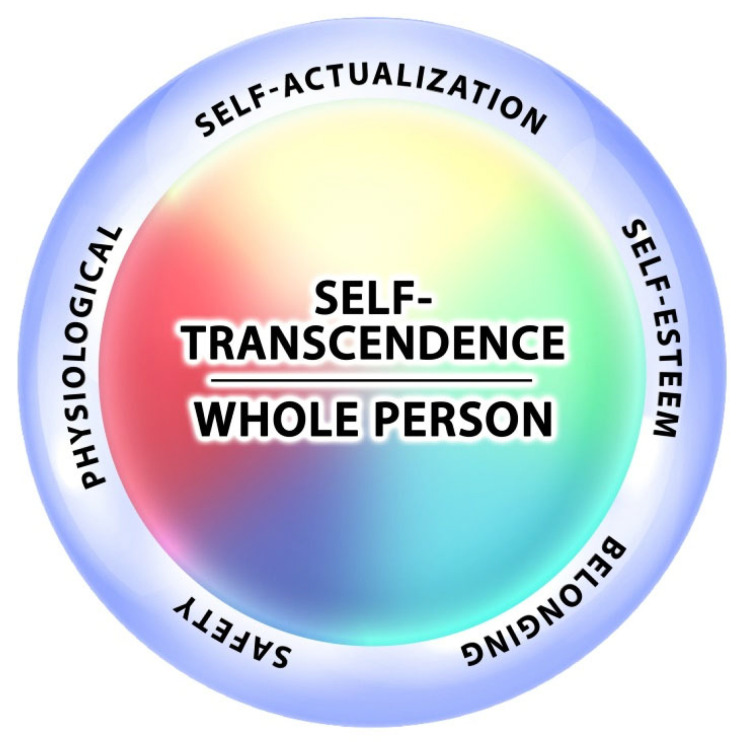
Maslow’s addition of self-transcendence, Maslow re-visited his work and added one more need, self-transcendence—the peak experience of the whole person.

Maslow revisited his own work on several occasions and as shown in Figure 4, he eventually added one more need that encompasses everything—self-transcendence [41]. This reflects a person’s ability to experience themself as a whole person, meeting multiple needs and values at the same time.

It is essential to recognize how core values play a major role in people’s emotional lives, including their work. For example, people’s commitment at work can be looked at as both rational and emotional. Rational commitment is about investing time, talent, and energy into the job at hand. Emotional commitment is based upon core values, such as dignity and respect. Phillips and Edwards found that emotional commitment is four times as valuable as rational commitment at work [16]. In a similar way, people’s motivations can be seen as external and internal. External motivations originate from outside oneself, such as salary and job promotion. Many regulations are often presented as external motivations. The problem is that external motivations are difficult for people to sustain over time. However, internal motivations arise from people’s core values. They carry a person through successes and failures, and the behaviors they generate can be sustained over a long period of time. Wrzesniewski and Schwartz found that people who are driven mostly by their internal motivations have the greatest success in reaching their goals [15].

People can feel pride and other strong emotions in their work, which can provide incredible commitment and motivation for each person. However, the emotional pain and disappointment of not being seen or heard, of not meeting one’s core values of dignity and respect, greatly decreases personal commitment and motivation. As a result, personal excellence, job satisfaction, personal excellence, and work quality can suffer [38].

#### 4.1.3. Work Culture and Community

Work culture is about how people experience their work, and ideally, how people can flourish in their work. Values themselves do not stand alone but exist and arise out of people’s cultures and communities. An organization’s structure, practices, and polices must serve and support these values, while each worker’s experience, desires, and growth reflect these values. When these values are met, the work culture succeeds and advances; when these values are not met, the needs and potential remedies of the culture become apparent [42].

To consider values within a work culture, it is important to consider the many ways that culture expresses itself. The United Nations Educational, Scientific and Cultural Organization (UNESCO) defines culture as... the set of distinctive spiritual, material, intellectual and emotional features of society or a social group, and…it encompasses, in addition to art and literature, lifestyles, ways of living together, value systems, traditions and beliefs. At work, two of these factors are essential—ways of living (and working) together, and value systems [17].

Looking at culture another way, Mkhize describes it more simply, noting that human beings cannot act without employing background knowledge—that knowledge that informs people’s decisions. This is culture [18]. This background knowledge remains intimate for each person; it is intrinsic and experienced and informs people’s decisions, both personal and professional. As a result, each person knows their culture, even if they cannot always articulate it. Workers will often demonstrate this background knowledge throughout their work and daily conversations [38].

People also express values through a sense of community. Communities can arise from the common ground of personal knowledge, a shared culture, and the values that individuals carry with them. Martin Mulligan and others described how community often begins as a sense of place at home or at work, but then grows beyond location to include relationships where people come together. This sense of community eventually grows into a way-of-life—with practices and traditions that people hold as common values [43]. Community as a way-of-life is not static—it is alive and dynamic. It grows and changes as people learn and grow, expanding their understanding of themselves and the world in which they live and work. When the workplace becomes community as a way-of-life, people can discover the relationships, knowledge and values that evolve into and form the foundation of a creative and growing work culture [38].

#### 4.1.4. Meaningful Work

Work is not who people are. However, in its most ideal setting, work can be an opportunity for people to express who they are. Indeed, work is one way that they bring themselves to the world. Work is also an opportunity for people to discover, know, and understand themselves through their actions and relationships. In this way, work helps them to build skills so that they can bring themselves even further to the world [42].

Core values have been shown through multiple surveys to be an important factor in what makes a job meaningful. Two, large surveys in the US and the UK found that jobs with a degree of professional independence and that make a direct, social contribution are the most meaningful and worthwhile [19]. Workers’ aspirations for their job—what they desire most for their job—is work that is meaningful, is under a supportive leadership, offers a positive work atmosphere, and brings good, social relationships. The young generation most often cites these qualities as major elements for their choice of work. They also look for support from their supervisor, a sense of independence, flexibility, and professional recognition [20]. Protecting the environment and fulfilling social responsibility by their employer are additional factors that companies use to attract talented young people [44].

#### 4.1.5. New Approaches in Leadership

Large organizations need certain dynamics to sustain themselves—therefore they develop structure, some form of hierarchy, and conformity. The question is: What within this structured work environment helps people get to that individual breakthrough when the next, great, personal experience of values, insight, or excellence occurs that furthers their work? Personal breakthroughs are an individual process; however many organizations require a significant amount of conformity which can evolve into a regulated bureaucracy. As a result, an individual’s breakthrough and creativity are pushed aside.

A key technique for bringing values to the workplace is values leadership. Reicher and others have pointed out how the leader identifies and understands the values of the individuals and communities in order to lead them. At work, the leader becomes one with their staff. Managers and workers act as equal partners, where workers have the opportunity to lead managers [23]. Values leadership requires balance to succeed. The Whitehall II Study has shown that vertical, top-down management, when left unto itself, has the potential to be out-of-balance and to greatly increase stress simply due to the nature of hierarchy [45]. This leaves people at the bottom of the hierarchy feeling a loss of control, a loss of being able to contribute, and a loss of predictability. This in turn creates chronic stress and the potential increase in accidents and ill health.

However, the vertical dimension of top-down management can be balanced by the horizontal, integrated development of workers and the work community. Here, values leadership provides the physical and social resources that support workers’ values, culture, and community. Leaders can further support workers by building relationships between managers and workers and amongst workers, emphasizing one-on-one conversations and personal interactions [46]. Management becomes most effective when it celebrates workers’ community. This becomes an environment of well-being where workers not only survive, but also flourish. By celebrating workers, leaders have the opportunity to bring justice and dignity to the workplace.

#### 4.1.6. Tools and Solutions for Supporting Core Values

Though many efforts have traditionally addressed safety through the intellect and the objective world, the challenge now is to also address safety through the subjective world of human connections and values. This requires subjective tools. People reside in this subjective world as human beings in their relationships and community, where workers can bring their expertise, as well as their personal knowledge and culture to their work. As an example, to explore the value of dignity at work, one must go beyond what dignity looks like to include what dignity feels like.

Building relationships becomes crucial. When people build relationships, they build an environment where workers can influence their work. Bennett and Jessani have described how the intangibles of trust…and…friendships can be more potent than logic and more compelling than evidence [47]. Health and safety is an intensely social process that depends upon relationships. It relies upon vibrant partnerships, collaborations, and above all personal contact between the people who create safety programs and the people that use them.

Another key technique to discovering values is simply this…listening. People want to be heard, not necessarily agreed with, but heard. It provides recognition, validation, and respect. Mkhize has described how, “It is necessary to listen, listen, and listen again, not just with our five senses, but with a sixth sense—listening with your heart.” [18] Larry Littlebird, a Pueblo storyteller, has said that all people need to do is—“Be quiet, sit down, and listen” [48]. Yaso Nadaraja explained how to go to that place in-between—resting in-between cultures and individuals and listening to common values [49]. Ultimately, people are listening for the answer to that question—Why are we being safe? Here, safety is no longer limited to information and company requirements. Safety now becomes a personal, emotional, and psychological experience [35].

Several worldviews also emphasize the subjective experience of humans by prioritizing family, community, and relationships as the foundation for their cultures [50]. Hopi leaders guide people to consider—What are your relationships? Be good to each other. In many parts of Europe, the concept of solidarity, working with others for others, remains a key, subjective element of culture. Rantanen has pointed out how occupational health requires solidarity for the common good, “… making working conditions safer, healthier, and human for everyone” [51].

Therefore, the solution is not another highly objective program or initiative. Instead, the people can use the tools already available. It is simply a matter of being human at work—taking the time to ask, “How are you doing? Is there anything you need?” These are the everyday, human tools of communication, relationship, and people being able to contribute to their work.

### 4.2. Core Values Supported within Organizations and OSH

#### 4.2.1. Meaning of Work in a Changing Environment

Work is changing rapidly and drastically due to many factors. Technology and digitalization, artificial intelligence, and an explosion of online social networks have deeply modified the way people work. Slashing, the holding of multiple jobs at the same time has become more and more common. Uberization, the digital platforms that directly connect service providers and customers, has increased social, economic, and human problems. Teleworking, working at home, has exploded during the COVID-19 pandemic, and has had both positive and negative impacts. Awareness of invisible jobs and informal workers, such as domestic jobs, voluntary work, assistance to people in need, and other work, remains insufficient and needs to increase. All these changes influence the way people experience their work where core values play a key role.

#### 4.2.2. Global Perspective

The global approach developed in the field of OSH requires multiple, relevant partners—workers, leaders, social partners, human resources managers, decision-makers, and politicians. Their role has become more and more important as OSH visibility decreases [10]. Core values represent an essential component of the holistic approach needed today as demonstrated in the following examples:

- The WHO framework Healthy Workplace has at its center Ethics and Values which explicitly underlines their core importance [3].
- PAHO promotes health through the core value of kindness [52].
- Within ISSA, the Vision Zero program integrates the three dimensions of safety, health, and well-being at all levels of work [25]. Focused mainly on the technical and organizational aspects of health and safety, it is based on a vision, an ideal state, that will never be reached but can provide a clear direction for promoting values such as trust, transparency, fair leadership, commitment, and collaboration.
- The Total Worker Health™ program seeks to improve the well-being of U.S. workers by protecting their safety and enhancing their health, well-being, and productivity [26]. It recognizes that work acts as a social determinant of health and supports the importance of values.

Holistic approaches such as these are starting to develop in this new century; they represent progress by breaking the self-contained silos within OSH that were predominant in the past but still remain in some cases [53].

These global efforts require the field of management and human resources to develop a more comprehensive approach to this growing awareness of values. The development and maintenance of a culture filled with these human and personal qualities is becoming a priority for companies looking for societal progress [27]. Business schools are already demonstrating this evolution. For example, at INSEAD, the non-profit, international, business school, HC de Bettignies has developed a new approach to educate business leaders and enhance their responsible leadership and global vision. This method, Awareness, Vision, Imagination, Responsibility and Action (AVIRA), supports managers as they develop their business ethics and social responsibility [54].

#### 4.2.3. Academic Research Perspectives and Salutogenesis

In OSH, core values have not been scientifically described and investigated, other than the fundamental principles required of health and safety professionals such as those outlined in the ICOH Code of Ethics [28]. Although adequate working conditions that include values are not met for many workers in a lot of workplaces, this does not mean this theme is utopian. First, there are situations where these values are already promoted [55], and second, core values may be held as a vison, an ideal to strive for, for those working conditions that have not yet allowed these values to materialize.

As a result, these values must be included in the training and education of OSH professionals. For this reason, the ICOH Committee for Education created the working group, Core Values at Work. Opening the scope of OSH to this broad perspective of work is important because it promotes each individual’s health and well-being.

Similar to this, one finds salutogenesis, another dimension within OSH. This parallels the practice of medicine that detects, treats, and prevents diseases, focusing on factors that promote good health. Salutogenesis was developed by Aaron Antonowski, a sociologist whose research focused on people’s resilience in extremely difficult situations, such as the people who survived the concentration camps of the Second World War [12,56]. He found that these people looked for solutions to their problems and identified their own resources to cope with them. Ultimately, these people found that these hardships held meaning within their lives. Antonowski developed a theory called Sense of Coherence that identifies those qualities that build resilience and promote health. Among these factors, core values are essential and consistent with how work can be fulfilling.

#### 4.2.4. Challenges, Pitfalls, and Possible Solutions

As stated earlier, we are not considering in this paper values that do not align with ethical, positive, human, and constructive qualities. However, there are challenges to values-based management—building a positive framework for values development, building mutual confidence and trust between partners, and ultimately, putting values into practice. Other barriers can also arise—inadvertent mistakes, a lack in understanding, inconsistencies in practice, and more.

To meet these challenges, companies must remain vigilant as they establish new models of leadership. Even with the best intentions, managers or employees with good, morale values may make unethical decisions without even realizing it. This is called ethical blindness [31]. Other unethical decisions may be taken either by a lack of awareness or by competing pressures to please authority or place profit above all else [57]. Similarly, the harmful, conscious misuse of core values to increase productivity and profit by pressuring workers to work harder and faster can damage workers’ health and well-being [32]. Further discussion of these pressures is important but beyond the scope of this paper. Ultimately, all these challenges require care and awareness to ensure the success of honoring workers’ core values.

The International Organization for Standardization (ISO) gives guidance to businesses and organizations and has promoted the Core Subjects of Social Responsibility as an official standard for operating in a socially responsible way [33]. As a result, values have emerged in some cases as a marketing argument to improve a company’s image. This, too, has advantages and pitfalls. Some companies publish their corporate values in different forms such as Codes of Conduct, Code of Ethics, or Charter of Core Values. These codes help to explain and clarify the principles that govern an organization’s vision and mission. When these values are met, the work culture succeeds and advances [35]. However, when these values are not met and respected, detrimental effects may occur. The organization can lose the trust and confidence of its workers and its credibility with clients and suppliers [58].

However, there are potential solutions to meet these challenges. Some mindful businessmen have proposed specific practices. One example is the approach developed by Herbert J. Taylor in the 1940s. He proposed the Four-Way Test of the things people think, say, or do. A successful, business executive, Taylor set up policies that reflect high ethics and morals in business. As president of Rotary International from 1954–1955, he offered his Four-Way Test to this service organization that still uses it worldwide as a moral code [34]. It provides a simple, ethical guide for personal and business relationships when looking for a solution to a problem. The test consists of four basic questions: (1) Is it the truth? (2) Is it fair to all concerned? (3) Will it build goodwill and better friendships? and (4) Will it be beneficial to all concerned?

A greater awareness of these values is emerging due to other advancing factors such as the young generation’s aspirations for a better world that respects work, the environment, fair trade, minorities’ rights, and more. Global solutions to meaningful work, climate change, energy production, hunger, and migration, all call for solidarity because they cannot be solved individually. This overarching need offers an opportunity to promote core values collectively, providing progress towards a society with greater integrity and dignity.

**Figure 5:**
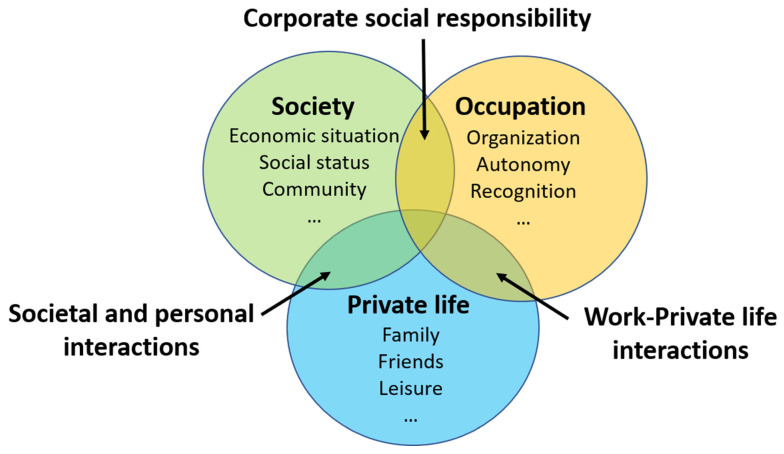
Examples of supports to core values in each sector of daily life. Each sector intersects and supports the others.

Figure 5 illustrates how society, the organization, and people’s private lives can come together in solidarity to form a picture of healthy individuals in a healthy society. If people see these potential solutions as a vision and an ideal to strive for, these interactions could become a driving force for people to work together for that ideal.

To promote core values in the workplace, there is an inescapable step: the introspection a person needs to understand their own core values, to think about what is ultimately important to them [59]. This understanding allows a worker to act with dignity and respect when creating healthy work conditions based on their core values. This can happen in many ways, through the individual, the organization, work practices, training, communication, and collaboration. By maintaining a clear intention throughout this process, with a clear understanding of one’s own values, a person can meet and overcome the challenges described above.

## 5. Conclusions

When people celebrate worker values, culture, and community, they have the opportunity to create work in its most ideal setting. Work can become an opportunity for people to express who they are, and to discover, know, and understand themselves. Work can become a means where workers not only protect each other’s bodies but help nourish each other to thrive emotionally and psychologically. For people to bring values to their work, it is important to honor the human context where their values reside. For they cannot be assigned to the intellect alone, but instead must also be honored in the personal and subjective realm of core values—the human, creative realm where people live, experience, and interpret their own personal lives.

## Figures and Tables

**Table 1:** Summary of the international ICOH sessions. These sessions were dedicated to spiritual and core values at work.

International Conference	Special Session Workshop	Chairpersons	Location Country	Reference
ICOH 31st—2015	Work and Spirituality	Max Lum	Seoul, South Korea	[5]
ICOH 32nd—2018	Work and Spirituality	Frank van Dijk Christophe Paris	Dublin, Ireland	[6]
Int. Conclave on OH 2020	Core Values at Work	Frank van Dijk	Mumbai, India	[7]

## Data Availability

This study did not report any data and is mainly based on a literature survey.
